# Effectiveness of reduction in conservative treatment of boxer fractures: A retrospective study of 115 patients

**DOI:** 10.1007/s00508-026-02711-1

**Published:** 2026-02-17

**Authors:** Paul Lennart Hoppe, Margarete Maria Steiner, Laura Maria Kurz, Thomas Haider, Stefan Hajdu, Gerhild Thalhammer

**Affiliations:** 1 Department of Orthopedics and Trauma Surgery, Medical University of Vienna, Währinger Gürtel 18–21, 1090 Vienna, Austria; 2 Department of Anaesthesia, Intensive Care Medicine and Pain Medicine, Medical University of Vienna, Währinger Gürtel 18–21, 1090 Vienna, Austria

**Keywords:** Boxer’s fracture, Radiological outcome, Casting, Closed reduction, Metacarpal fracture

## Abstract

**Purpose:**

Subcapital fractures of the fifth metacarpal bone are one of the most common fractures of the hand. Surgical and conservative treatment is possible but the respective indications remain controversial. Closed reduction and cast retention are commonly performed for these fractures but their effectiveness in fracture retention is unclear. Therefore, the aim of this study was to evaluate the efficacy of closed reduction with consecutive below-elbow casting and finger splinting.

**Methods:**

Radiographs of 115 patients with a subcapital fracture of the fifth metacarpal bone at an academic level 1 trauma center were retrospectively reviewed and volar angulation was assessed at initial presentation, after reduction and at the end of treatment.

**Results:**

The patients mean age was 32 years (IQR 25–37 years) and 93% were male (*n* = 107). Cast immobilization was 4 weeks in all cases. Volar angulation at initial presentation was 49.9° ± 11°. After reduction an angle of 40.9° ± 9.2°was observed. After cast removal, an angulation of 42.4° ± 9.9° was observed. A repeated measures ANOVA with a Huynh-Feldt correction showed that the difference in mean fracture angulation was statistically significant.

**Conclusion:**

This study indicates that improved fracture angulation can be maintained with closed reduction and retention in a below-elbow cast with finger splinting. Further studies have to be conducted to investigate whether improved fracture angulation results in more favorable clinical outcomes.

## Introduction

Subcapital fractures of the fifth metacarpal bone, also referred to as boxer fractures, are among the most common fractures of the hand [1–3], accounting for approximately 20% of all hand fractures. They usually result from axial loading of the clenched fist, as occurs during boxing, hence the name. This leads to a fracture of the weakest part of the metacarpal bone, the distal metaphysis.

When classifying subcapital fractures of the fifth metacarpal, the identification of neck versus shaft method is used. It defines this kind of fracture as fracture with ≥ 75% of the fracture line lying within a square formed by the C‑line that spans across the broadest part of the metacarpal head [4].

**Fig. 1:**
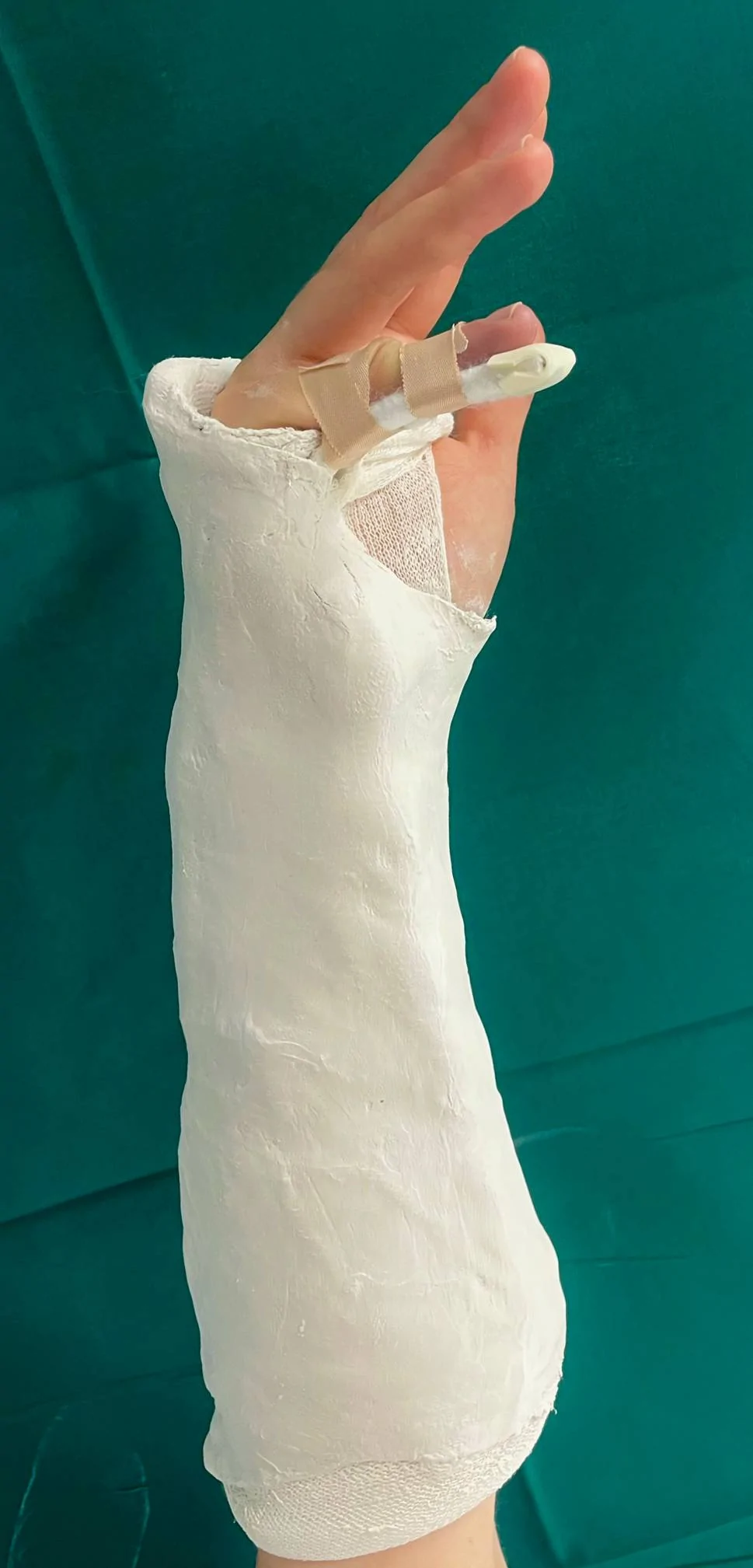
Below-elbow cast and finger splint after fracture reduction of a boxer fracture

In most cases these fractures can be treated conservatively with closed reduction and cast immobilization, even in cases with extensive fracture angulation; however, there is still a risk for secondary displacement. This occurs mainly due to the intrinsic muscles forcing the metacarpal head into palmar angulation through their attachment at the base of the proximal phalanx [5–7]. At our department patients are treated with a below-elbow cast and finger splint after fracture reduction using local anesthesia (Fig. 1). Reduction is typically achieved by the Jahss maneuver and retained by applying longitudinal traction, which is supposed to result in improved length and angulation [8].

Cut-off values of fracture angulation regarding indications for treatment remain controversially discussed. Most commonly angles of up to 50° are accepted for conservative treatment [7, 9], although some authors even tolerate angulations of up to 70° [10, 11]. When choosing surgical treatment, various methods are available. Most commonly antegrade intramedullary k‑wire fixation is performed due to its low complication rate, cost effectiveness, good results and minimal soft tissue injury [12–14]. Further indications for surgery include malrotation of the finger, leading to scissoring of the fourth and fifth fingers and potential comminution resulting in an unstable fracture [15].

A recent study on ulnar gutter casts has shown that fracture retention after initial closed reduction was not successful, as after 4 weeks no improvement in fracture angulation was observed [16]. Furthermore, at international congresses, expert opinion has raised the question of whether fracture reduction may even lead to destabilization of boxer fractures, leading to increased dislocation after all [17]. To date, biomechanical data supporting this hypothesis are lacking.

In our study we aimed to evaluate if after reduction an improved fracture angle can be maintained with conservative treatment using a below-elbow cast and finger splint or if this leads to destabilization, resulting in a worse outcome.

## Methods

This study was performed at the Department of Orthopedics and Trauma-Surgery at a level 1 trauma center. All procedures followed were in accordance with the ethical standards of the responsible committee on human experimentation (institutional and national) and with the Helsinki Declaration of 1975, as revised in 2008. The study was approved by the local Ethics Committee (no. 1092/2024) on 15 May 2024, with the need for written informed consent waived.

**Fig. 2:**
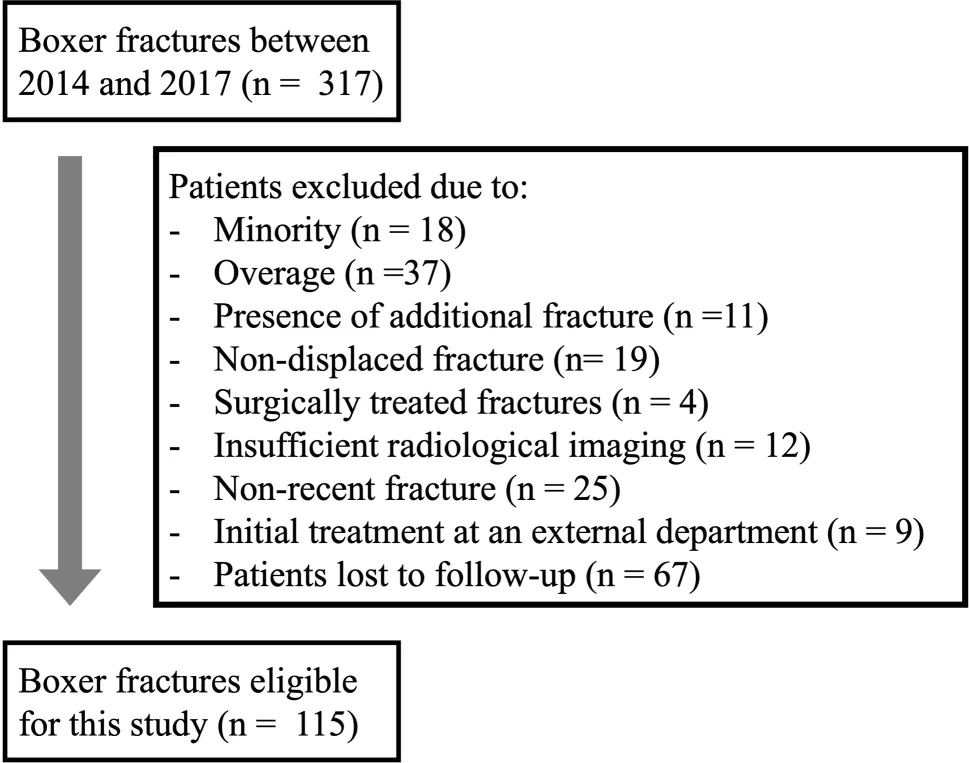
Flow chart of patient selection

Data collection was done retrospectively from the local patient database, including all patients with subcapital fractures of the fifth metacarpal bone between 2014 and 2017. In total, 317 patients with boxer fractures were treated at our department during this time frame. Exclusion criteria were the following: patients under the age of 18 years at the time of diagnosis, patients older than 65 years at the time of diagnosis, presence of additional fractures in the injured hand, nondisplaced fractures, surgically treated fractures, insufficient radiological imaging, fractures older than 7 days at initial presentation, initial treatment at an external department, conservatively treated fractures with casting for less than 3 weeks and patients lost to follow-up (Fig. 2).

All patients were treated with a below-elbow cast augmented with a rigid aluminium finger splint. Prior to reduction and casting local anesthesia was injected. Either a hematoma block or proximally injected Oberst’s block was chosen, depending on the surgeons’ preference. Primary reduction was performed using the Jahss maneuver.

The aluminium finger splint was bent according to the patients’ anatomical needs, in order that the finger was held in an intrinsic plus position and with correct rotation. To achieve this and to be able to apply longitudinal traction, the splint has to bend slightly proximal to the metacarpophalangeal (MCP) joint. The splint was then fixated on the patients forearm and hand, the fifth finger was not yet placed on the splint. Then a dorsal plaster longuette was applied and was only circularly closed on the second day after injury, if the swelling allowed this. The finger was placed on the splint under continued traction after retaining the reduction by applying longitudinal traction. It was then secured on the splint using two tape strips, one was wrapped around the proximal phalanx and one around the middle phalanx.

Radiographs after reduction and casting were performed to evaluate efficacy of reduction and fracture alignment. If necessary, reduction and cast application was repeated.

Follow-up visits at the outpatient clinic were scheduled 7 and 14 days after trauma, to assess cast condition and efficacy, as well as to perform X‑ray imaging. If necessary, finger fixation or the whole cast were renewed but without repetition of closed reduction maneuvers. In cases of loss of reduction with a fracture angle greater than 50° surgical intervention was recommended to the patient. After 4 weeks of immobilization the fracture was assumed to be healed, the cast was removed, and final X‑ray imaging was conducted.

**Fig. 3:**
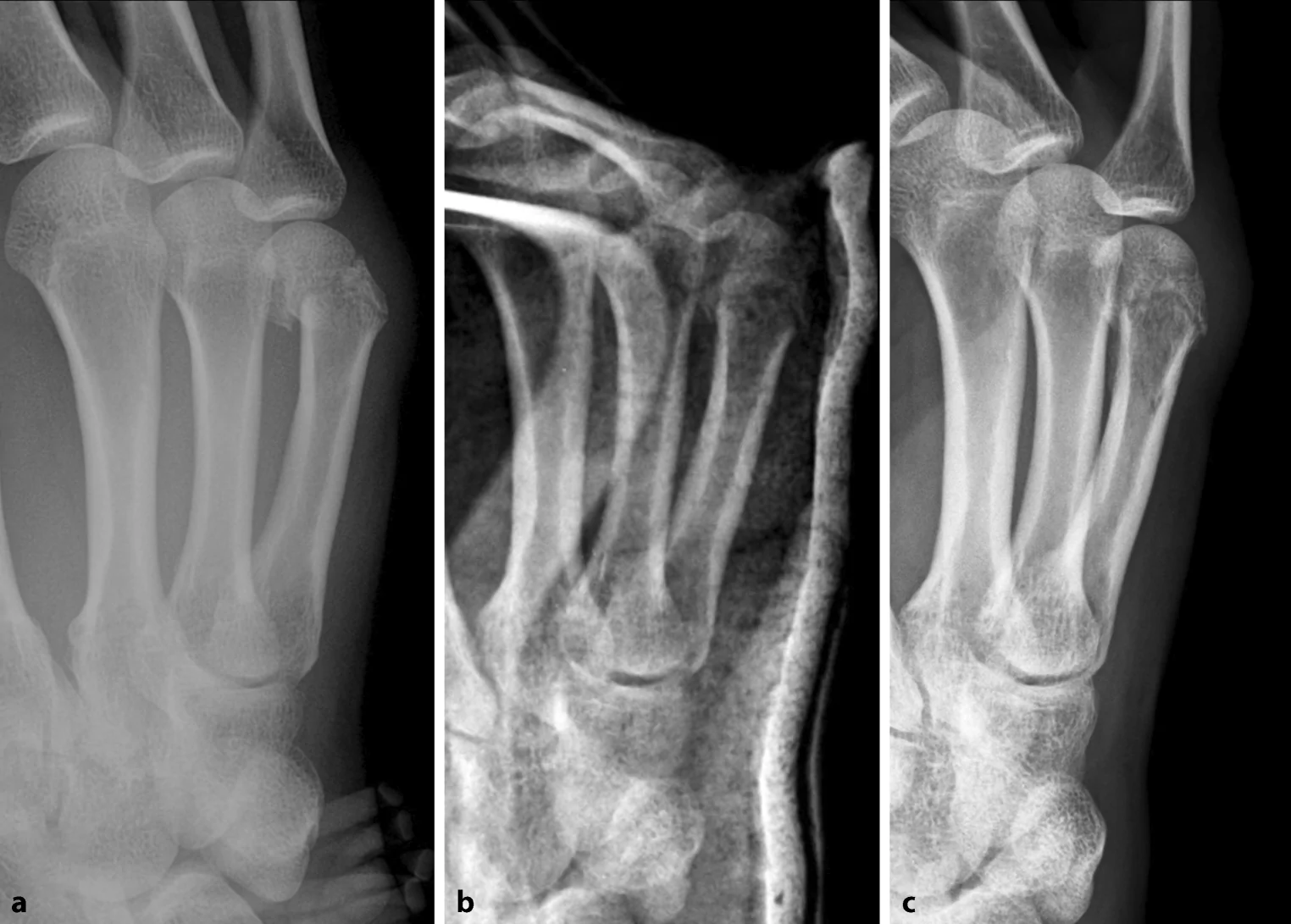
Radiographs of the fingers of a single patient during course of treatment—at initial presentation after trauma (**a**), with noticeable reduction in the cast (**b**) and consolidated in improved fracture angulation (**c**)

In all patients, volar angulation was assessed in X‑ray images obtained at initial presentation in the emergency room, after reduction and cast application as well as after cast removal at the end of treatment (Fig. 3). The medullary canal-oblique (MC-30) measuring method, as previously described by Sletten et al., was used to determine volar angulation. This method uses X‑ray images that are taken at an oblique angle of 30° [18–20]. The first line is drawn centrally through the medullary canal of the metacarpal bone, while the second line is drawn from the mid-medullary point in the center of the neck fracture to the most distal point of the metacarpal head. The angulation between these two lines is then measured.

Statistical analysis was performed using IBM SPSS Statistics Version 29, 64-bit (IBM, Armonk, NY, USA). Normal distribution was assessed by the analysis of histograms. Subsequently, repeated measures analysis of variance (ANOVA) was performed to detect any differences in volar angulation at different times of treatment. A violation of sphericity was detected, therefore the Huynh-Feldt adjustment was used for correction. For normally distributed data the arithmetic mean and standard deviation were calculated, while for non-normally distributed data the median and interquartile ranges were calculated. *P*-values lower than 0.05 were considered as statistically significant.

## Results

In total, 115 patients were included in this study. Mean age was 32 years (IQR 25–37 years) and with 93% the majority of patients were male (*n* = 107). In 83 cases (72%) fractures affected the right hand and 1 patient in the study population presented with an initial malrotation deformity. Closed reduction successfully restored anatomical alignment in this patient. Only four patients were excluded due to surgical treatment involving bouquet pinning. In all cases, the indication for surgery was the patient’s preference for anatomical alignment to preserve the physiological knuckle contour for esthetic reasons. In all patients, closed reduction improved fracture angulation to less than 50°, eliminating the need for surgical intervention based on biomechanical considerations. No patient underwent secondary surgery after bony consolidation to improve fracture alignment. All included patients underwent standardized radiographs of the hand in 30° oblique view and anteroposterior (AP) view at every outpatient clinic visit.

**Fig. 4:**
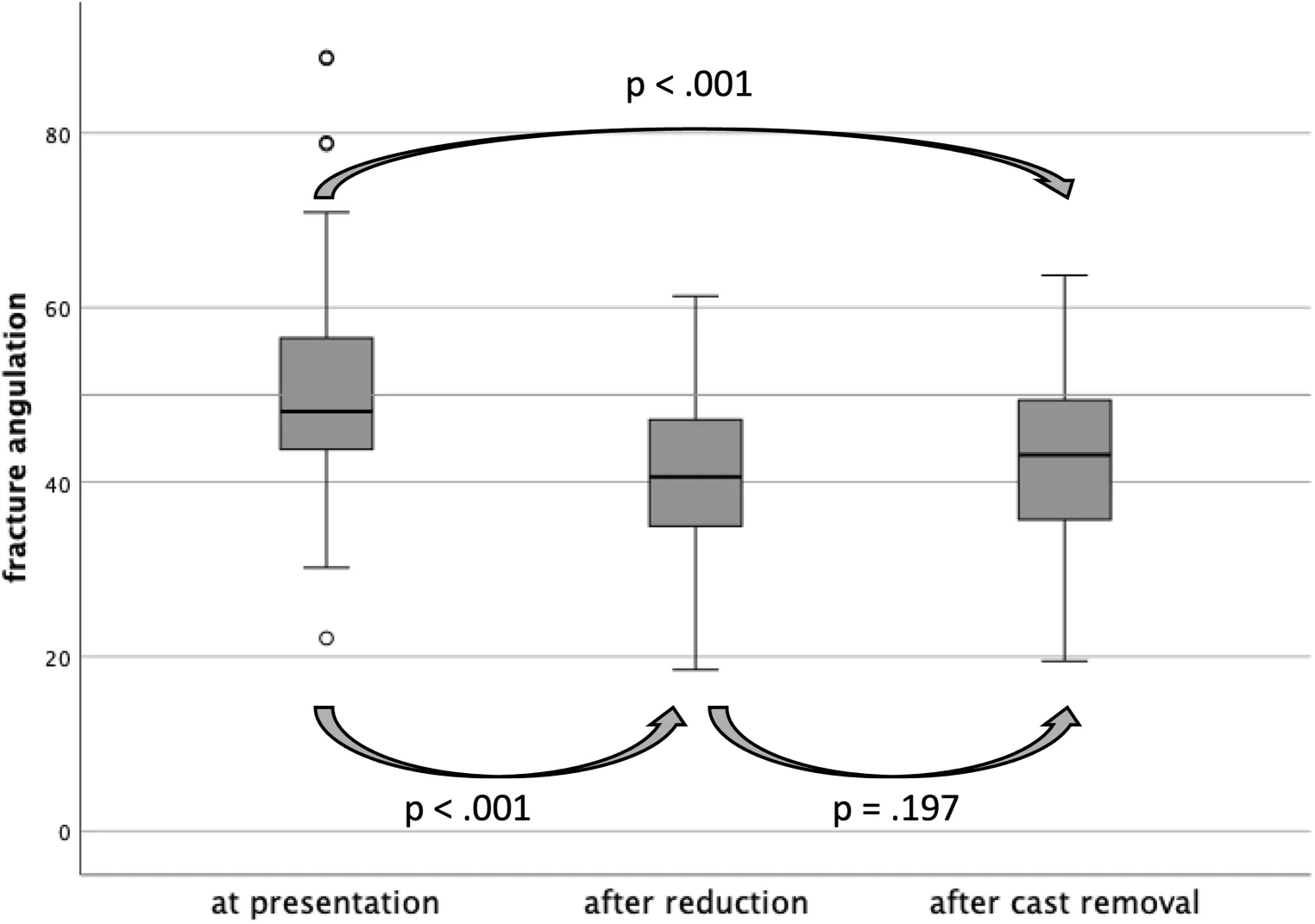
Boxplots of fracture angulation in degrees during course of treatment, with specified statistical significance

Volar angulation at initial presentation was 49.9° ± 11° (95% CI, 47.9°–51.9°), after reduction a fracture angulation of 40.9° ± 9.2° (95% CI, 39.2°–42.6°) was achieved. The mean duration of immobilization was 28.1 ± 2.9 days. After cast removal, an angulation of 42.4° ± 9.9° (95% CI, 40.6°–44.2°) was documented. A repeated measures ANOVA with a Huynh-Feldt correction showed that the difference in mean fracture angulation was statistically significant, F (1.74, 198.71) = 43.74, *p* < 0.001 (Fig. 4). Bonferroni-adjusted post hoc analysis also revealed a statistically significant decreased fracture angulation after reduction and cast removal compared to the initial X‑rays (*p* < 0.001). No statistically significant secondary dislocation between postreduction and final radiographs was observed (*p* = 0.197).

## Discussion

In this retrospective study, we were able to show a statistically significant improvement in fracture angulation with closed reduction and cast immobilization with a finger splint. The mean improvement of fracture angulation between initial presentation and final follow-up was 7.5°. Until now, there have been no other studies available evaluating the efficacy of conservative treatment in terms of below-elbow cast and finger splint; however, previous studies have examined whether improved fracture angulation can be maintained following closed reduction and casting with an ulnar-gutter cast. Pace et al. performed a retrospective study to compare the efficacy of the ulnar gutter cast with or without prior closed reduction in a case-control study. They did not find a statistically significant difference in fracture alignment, neither between the initial radiograph after trauma and at the end of treatment nor between both groups [16].

Van Aaken et al. compared in a prospective randomized study the MCP extension cast with closed reduction, to soft wrapping and buddy taping without reduction [9]. Duration of cast treatment was also 4 weeks. They observed a statistically significant improvement in angulation after reduction but also a statistically significant loss of reduction during the course of cast treatment. Nevertheless, after 4 months an improved fracture angulation from initially 53° ± 13° to 45° ± 9° was observed. A significant secondary loss of reduction following closed reduction and immobilization in an ulnar gutter cast in the intrinsic-plus position has likewise been described in the literature [21].

This might mean that there is a difference in the efficacy of retaining reduction between different cast types. So far, no studies have compared the ulnar gutter cast or the MCP extension splint with the below-elbow cast with finger splint. Taking these different studies into account, boxer fractures can be sufficiently reduced and retained depending on the treatment regimen. Destabilization of the fracture due to closed reduction resulting in worse fracture alignment was not observed, neither in our study nor in the cited studies.

To achieve a truly anatomical alignment in these fractures, surgery is most often necessary [22]. Sletten et al. compared the outcome of conservative treatment versus antegrade intramedullary bouquet pinning in a prospective multicenter randomized controlled trial [4]. The initial median palmar angulation in the group treated operatively was 40° (30–59°) and improved to a median of 17° (9–31°) angulation after surgery. In the conservatively treated group, the initial fracture angulation was 41° (30–58°) but angulation at the end of treatment was not reported. Nevertheless, no statistically significant clinical outcome differences were shown in this study. The authors therefore recommended conservative treatment for fractures with angulations of up to 50°. A similar study with nearly the same outcome was performed by Strub et al. [23].

It must be noted that biomechanical studies have shown a decrease in grip strength in patients with fractures angulated more than 30° [24, 25]; however, clinical studies were not able to confirm a significant reduction in grip strength after conservative treatment [7, 26]. Therefore, greater angulation and potentially reduced grip strength do not appear to be clinically significant or noticeable to patients.

We were able to show that improved fracture angulation can be maintained with conservative treatment; however, studies have shown similar results for fractures treated with reduction and early functional immobilization protocols like the metacarpal brace [27]. A comparison between reduction and below-elbow cast with finger splint, reduction and metacarpal brace as well as early functional treatment without immobilization still has to be performed. Previous studies have shown that when early functional treatment without immobilization is compared with cast immobilization with or without prior reduction, a significant improvement in the functional outcome is observed in the group receiving early functional treatment [9, 10, 28–30]; however, these studies vary in quality and most of them are reported to appear unsatisfactory upon critical evaluation [31].

It has to be acknowledged that truly accurate measurements of fracture angulation are rare, as strictly lateral X‑rays are seldomly performed, although this view would enable the most accurate evaluation of angulation. Nevertheless, X‑rays are most commonly performed in 30° pronation, as this view results in less overlapping bone structures and therefore enables a more reliable diagnosis and lower risk of missing fractures [18, 20]. This is also the standard in our clinic, meaning that for this study 30° oblique X‑ray images were examined. More precise measurements of angulation could be obtained by using strict lateral radiographs.

In our study we observed a high exclusion rate due to loss to follow up. This is a common issue in metacarpal fractures, with reported follow-up nonattendance rates of up to 30% after 1 month [32]. Regarding patient characteristics, several authors [4, 33, 34] have shown that boxer’s fractures predominantly occur in young males with a median age between 22 and 32 years. Our patient population is therefore consistent with findings in the existing literature.

Further limitations of this study arise from the retrospective character and the isolated examination of radiographs of patients treated with a cast and finger splint. In finding the relevance of improved fracture angulation, patient-reported outcome measurements and assessments of range of motion and grip strength are necessary. These parameters were not included in this study but warrant further studies.

## Conclusion

This study was performed retrospectively to evaluate the effectiveness of closed reduction and consecutive conservative treatment in patients with boxer fractures, with respect to fracture angulation and secondary dislocation. We were able to show a statistically significant improvement in fracture angulation between initial presentation and after cast removal at the end of conservative treatment.

## Data Availability

The data that support the findings of this study are available from the corresponding author upon reasonable request.
